# Inflammatory and fibrotic signaling pathways mediated by cardiac macrophages in atrial fibrillation

**DOI:** 10.3389/fcvm.2025.1692638

**Published:** 2026-01-05

**Authors:** Haoqing Ren, Hengli Lai, Zhenhuan Chen

**Affiliations:** 1 Jiangxi Medical College, Nanchang University, Nanchang, China; 2 Department of Cardiology, Jiangxi Provincial People’s Hospital, The First Affiliated Hospital of Nanchang Medical College, Nanchang, China

**Keywords:** atrial fibrillation, cardiac macrophages, atrial remodeling, inflammation, fibrosis

## Abstract

Atrial fibrillation (AF) is traditionally characterized as an electrophysiological disorder; however, growing evidence underscores its intimate connection with immune dysregulation, particularly inflammation-driven structural remodeling. This review aims to comprehensively elucidate the role of cardiac macrophages in AF pathogenesis, focusing on their involvement in inflammatory and fibrotic signaling, electrical remodeling, and intercellular interactions. By systematically reviewed previous studies, this reviewing summarises how macrophages act as central modulators of AF through phenotype-specific mechanisms. M1-polarized macrophages contribute to electrical instability by releasing pro-inflammatory cytokines that affect ion channel expression and action potential duration. In contrast, M2 macrophages promote fibroblast activation and collagen deposition transforming growth factor-beta 1(TGF-*β*1), interleukin-10 (IL-10), and Tumor Necrosis Factor Superfamily Member 14 (LIGHT) signaling, leading to atrial fibrosis. Evidence from human samples, animal experiments, and transcriptomic data converge on macrophage density, polarization state, and cytokine signatures as key correlates of AF severity and recurrence. Targeting their activation states and signaling pathways represents a promising avenue for mechanism-guided AF therapy. Therefore, this review provides a consolidated framework for future translational strategies aiming to interrupt the immune-mediated remodeling cascade in AF.

## 1 Introduction

Atrial fibrillation (AF) is the most prevalent sustained cardiac arrhythmia, affecting over 60 million individuals globally, with its incidence continuing to rise in parallel with population aging and the increasing prevalence of cardiometabolic risk factors (1). Epidemiological data indicate that approximately one in three adults in the United States will develop AF in their lifetime (2). The Global Burden of Disease Study also noted that disability-adjusted life years (DALYs) attributed to AF more than doubled from 3.8 million in 1990 to 8.4 million in 2019, emphasizing its increasing public health relevance (1, 2). This trend underscores AF not only as a growing clinical challenge but also as a critical public health issue that requires intensified efforts in early prevention, risk stratification, and targeted intervention strategies (3).

Despite the multifactorial etiology of AF, conventional mechanistic models have primarily emphasized three interrelated processes: electrical remodeling, structural remodeling, and autonomic remodeling (4). Electrical remodeling typically refers to action potential duration (APD) shortening and effective refractory period (ERP) reduction, largely mediated by downregulation of L-type calcium currents and alterations in potassium channel expression, which facilitate reentrant circuits and arrhythmogenesis (5). Structural remodeling, on the other hand, encompasses atrial dilatation, myocyte hypertrophy, and interstitial fibrosis, which impair conduction continuity and promotes the substrate for reentry (6). During this process, the autonomic nervous system further contributes through sympathovagal imbalance, which destabilizes the electrophysiological properties of atrial tissue (7). However, growing evidence now implicates the immune system as a critical contributor to both the initiation and perpetuation of AF (8). Clinical and preclinical studies have demonstrated that elevated levels of circulating inflammatory cytokines, such as Interleukin-6 (IL-6), Interleukin-1 beta (IL-1β), and Tumor Necrosis Factor-alpha (TNF-α), correlate with AF burden and recurrence (9). In addition, cardiac macrophages, especially when polarized to proinflammatory M1 phenotypes, have been shown to modulate calcium channel expression in atrial myocytes and facilitate fibrotic signaling cascades through interactions with fibroblasts (10–12). Moreover, histological analysis of atrial tissue from AF patients has revealed increased infiltration of CD68^+^ macrophages and upregulation of immune checkpoint and inflammasome markers, further supporting the integration of immune-mediated inflammation into the classical AF remodeling paradigm (13, 14).

Beyond classical paradigms of electrical and structural remodeling, emerging studies have proposed the concept of immune remodeling to characterize the sustained pro-inflammatory state and immune cell dysregulation that underlie AF persistence (13). This immunological dimension of remodeling is increasingly recognized as a distinct and parallel pathophysiological axis, in which innate immune cells, particularly cardiac macrophages, play a central role in bridging inflammatory signaling and tissue remodeling (8). Cardiac macrophages constitute a heterogeneous population of resident and infiltrating cells that dynamically respond to stress cues within the atrial microenvironment (15, 16). Furthermore, the interplay between cardiac macrophages and atrial fibroblasts establishes a paracrine amplification loop, enhancing structural disarray and conduction heterogeneity (12). This immuno-fibrotic coupling positions macrophages as a key factor in atrial remodeling. Therefore, immune remodeling through macrophage activation and phenotypic transitions has emerged as a pivotal component of the arrhythmogenic milieu in AF and represents a promising target for disease-modifying interventions.

Given the emerging paradigm of immune remodeling in AF, there is an urgent need to systematically delineate the cellular mechanisms and molecular pathways through which cardiac macrophages contribute to disease initiation and progression. Despite increasing recognition of their roles in modulating inflammation and fibrosis, current understanding remains fragmented, with limited integration of spatiotemporal evidence across experimental models and clinical observations. This review therefore aims to provide a comprehensive synthesis of the inflammatory and fibrotic signaling pathways mediated by cardiac macrophages in the context of AF. We highlight key upstream activators, macrophage phenotypic transitions, downstream effector molecules, and their interactions with other atrial cell types. By clarifying the immuno-inflammatory basis of atrial remodeling, this review contributes to the evolving conceptual framework of AF pathophysiology and supports the development of macrophage-centered therapeutic strategies.

## 2 Cardiac macrophage activation and phenotypic polarization in AF

Accumulating evidence suggests that cardiac macrophages are not a static population but rather exhibit remarkable heterogeneity and plasticity in response to pathological stimulus (13, 15, 17). In the context of AF, these cells undergo dynamic recruitment, activation, and phenotypic reprogramming, which collectively shape the local inflammatory milieu and fibrotic landscape of the atrial myocardium. Understanding the polarization trajectory of macrophages within atrial tissue is thus fundamental to elucidating how immune remodeling integrates with electrical and structural remodeling during AF development and maintenance.

### 2.1 The origin and recruitment mechanism of cardiac macrophages

Cardiac macrophages in both physiological and pathological conditions originate from two major sources (16). The first comprises embryonically derived resident macrophages, which arise from the yolk sac or fetal liver and are maintained through self-renewal in the adult heart. The second population is replenished by bone marrow–derived monocytes that are recruited to the myocardium during injury or inflammation, subsequently differentiating into inflammatory macrophages.

In the setting of AF, the atrial tissue exhibits a pronounced inflammatory state, characterized by the robust recruitment of circulating monocytes into the atrial endocardium and interstitium (18). This process is primarily orchestrated by several chemokines, including monocyte chemoattractant protein-1 (MCP-1/CCL2), macrophage inflammatory protein-1*α* (MIP-1*α*/CCL3), and macrophage colony-stimulating factor (M-CSF). Among these, the MCP-1/CCR2 axis plays a pivotal role in monocyte mobilization from the periphery into the heart (19). Furthermore, hypoxic conditions, commonly present in AF-related comorbidities such as obstructive sleep apnea, can induce HIF-1*α* expression in atrial cardiomyocytes, promoting the upregulation of macrophage migration inhibitory factor (MIF) (10, 20). MIF binds to its receptor. Cluster of Differentiation 74 (CD74) on macrophages, activating the Nuclear Factor kappa-light-chain-enhancer of activated B cells (NF-*κ*B) signaling pathway, thereby enhancing M1-type polarization and maintaining local pro-inflammatory properties (10).

Additionally, in mouse models of AF induced by chronic pressure overload or rapid atrial pacing, a significant increase in F4/80^+^/CCR2^+^ monocyte and macrophage infiltration has been observed. These infiltrating macrophages are closely associated with atrial conduction slowing and increased arrhythmogenicity (14, 21, 22). Notably, targeted depletion of CCR2^+^ cells alleviated atrial fibrosis and reduced AF inducibility, underscoring the functional contribution of recruited macrophages to the disease process (22). Moreover, inflammation-mediated changes in the atrial microenvironment enhance interferon pathways and chemotactic signals, further sustaining monocyte recruitment and differentiation. These events amplify the local macrophage population and promote their pathogenic roles in inflammation, fibrosis, and electrical remodeling (8).

In summary, atrial macrophages in AF derive from both resident populations and recruited circulating monocytes. Their recruitment and expansion are tightly regulated by chemokines, hypoxia-inducible factors, and cross-talk with atrial structural cells. These mechanisms represent a critical immunological axis that initiates and sustains atrial immune remodeling and offer potential targets for therapeutic intervention.

### 2.2 Macrophage subtypes and polarization spectrum

Cardiac macrophages in the atrial tissue exist in multiple phenotypic states, driven by local cues such as hypoxia, oxidative stress, and neurohormonal factors, rather than fitting strictly into the classical M1 (pro-inflammatory) or M2 (anti-inflammatory) categories. While M1 macrophages are characterized by the production of pro-inflammatory cytokines like IL-1β, TNF-α, and Inducible Nitric Oxide Synthase (iNOS) and are typically induced by stimuli like interferon-*γ* (IFN-*γ*) or lipopolysaccharide (LPS) (23). In contrast, M2 macrophages exhibit anti-inflammatory and reparative phenotypes, commonly induced by interleukin-4 (IL-4) and interleukin-10 (IL-10). These cells express CD206 and secrete fibrotic mediators such as TGF-*β*1, osteopontin (OPN), and arginase-1 (Arg1), which promote fibroblast activation and extracellular matrix deposition, key processes in atrial structural remodeling (24, 25). However, recent evidence reveals that macrophage polarization in AF is highly plastic and exists along a spectrum. Instead of being restricted to M1 or M2, macrophages can co-express M1 and M2 markers simultaneously or transition between these states in response to evolving local cues such as hypoxia, oxidative stress, and neurohormonal factors (23, 26). This phenotypic plasticity allows macrophages to mediate inflammatory responses and initiate fibrotic repair simultaneously, depending on the temporal and spatial context.

Single-cell RNA sequencing (scRNA-seq) of atrial tissues further confirms the coexistence of mixed phenotypes and intermediate macrophage subtypes, some of which do not fit the classical M1/M2 paradigm but rather express unique profiles related to antigen presentation, tissue remodeling, or metabolic activation (27, 28). These intermediate macrophages may be key players in the chronicity and persistence of AF, acting as modulators of both acute injury and long-term fibrosis (29). Therefore, understanding macrophage polarization in AF should go beyond the classical M1/M2 dichotomy and consider multiple activation states based on gene expression signatures rather than relying solely on surface markers (15). Key activation states in AF include an IL-1β–NLRP3-driven inflammatory phenotype, a TGF-*β*/OPN-dominant fibro-inflammatory phenotype, a reparative phenotype involved in matrix turnover, and an interferon/antigen-presenting phenotype involved in adaptive immune signaling (12, 22).

Emerging regulatory layers also include microRNA-mediated modulation of macrophage differentiation. Recent work has shown that miR-363-5p, together with IL-34 signaling, can influence macrophage functional states and indirectly affect cardiomyocyte electrophysiology by modulating pacemaker channel HCN4 expression (30). Although these findings were derived from iPSC-derived cardiomyocyte models, they highlight a broader post-transcriptional regulatory framework that may intersect with macrophage polarization in atrial remodeling.

**Figure 1:**
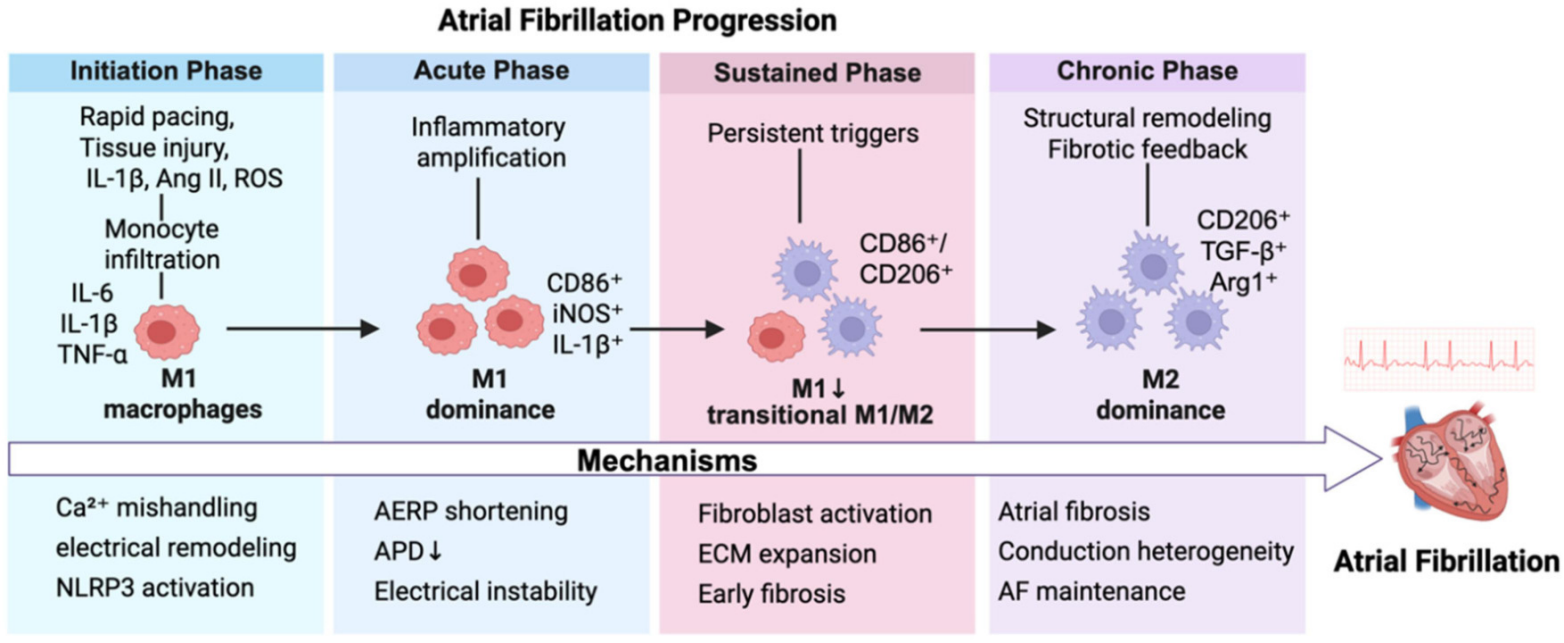
Temporal progression of macrophage-mediated mechanisms in AF. This diagram illustrates the evolution of AF across four stages:Initiation, Acute, Sustained, and Chronic phases. In the initiation phase, rapid atrial pacing, tissue injury, and oxidative stress trigger monocyte infiltration and polarization into M1 macrophages (IL-6^+^, IL-1β^+^, TNF-α^+^), contributing to calcium mishandling, early electrical remodeling, and NLRP3 activation. During the acute phase, M1 macrophage dominance leads to amplified inflammation and action potential duration (APD) shortening. The sustained phase features a transition toward mixed M1/M2 phenotypes (CD86^+^/CD206^+^), driving extracellular matrix expansion and early fibrosis. In the chronic phase, M2 macrophages (CD206^+^, TGF-*β*^+^, Arg1^+^) dominate, promoting fibrosis, conduction heterogeneity, and AF perpetuation through structural remodeling.

Figure 1 summarizes the temporal evolution of cardiac macrophage subpopulation conversion, secreted factor profiles, and related mechanisms during the critical stages from AF onset to the chronic phase, further revealing their central role in disease progression.

### 2.3 Macrophage and immune cell interactions in atrial remodeling

Macrophages are pivotal in driving immune-mediated atrial remodeling, but they do not act in isolation. Recent evidence points to substantial interactions between macrophages and other immune cell types, notably T cells, neutrophils, and B cell, which together shape the inflammatory, fibrotic, and electrophysiological substrate of the atria (8). For example, elevated infiltrating CD4^+^CD28^−^ T cells in AF correlate with increased macrophage recruitment and TNF-*α*/IFN-*γ* release, creating a pro-inflammatory milieu that amplifies NLRP3 activation in macrophages (14, 31). Neutrophils and their released neutrophil extracellular traps (NETs) can activate macrophages and fibroblasts, promoting atrial fibrosis and conduction heterogeneity (18). In parallel, B-cell derived autoantibodies may modulate macrophage Fc-receptor signaling and alter their polarization toward a fibro-inflammatory phenotype.

Together, these inter-cellular networks create a self-reinforcing immune-remodeling loop: T and neutrophil signals drive macrophage activation, macrophages further recruit and modulate adaptive immune cells, and this cross-talk underpins the persistence and progression of AF.

## 3 Inflammatory and fibrotic signaling pathways mediated by cardiac macrophages

Macrophages play a pivotal role in the progression of AF by not only participating in immune cell infiltration and structural remodeling but also releasing various inflammatory and fibrotic cytokines, thereby activating a series of signal transduction pathways, and thereby exerting a central role in the development of myocardial electrophysiological instability and interstitial fibrosis. Different phenotypes of cardiac macrophages can regulate fibroblast activity, myocardial cell ion channel expression, and matrix metabolic balance through paracrine effects, forming a cross-cellular, cross-process “inflammation-fibrosis-electrical” coupling network.In recent years, several key factors and pathways have been identified as playing decisive roles in this process, including the IL-1β–QKI–CACNA1C axis, NLRP3 inflammasome–ROS signaling, and TGF-*β*–Smad pathways, revealing the multidimensional regulatory potential of cardiac macrophages in the pathological progression of atrial fibrillation (11, 22, 24).

### 3.1 IL-1β–QKI–CACNA1C pathway

Interleukin-1β (IL-1β) has been identified as a central effector molecule secreted by activated macrophages (9, 32). Accumulating evidence demonstrates that IL-1β downregulates the expression of the RNA-binding protein Quaking (QKI) in cardiomyocytes, disrupting post-transcriptional control of ion channel transcripts. QKI has been shown to bind and stabilize Calcium Voltage-Gated Channel Subunit Alpha1 C (CACNA1C) mRNA, which encodes the *α*1C subunit of L-type calcium channels (ICa-L), a component integral to maintaining atrial action potential duration (21). Experimental models of inflammation and myocardial injury reveal that IL-1β exposure leads to a marked suppression of QKI mRNA and protein levels in atrial cardiomyocytes, resulting in decreased CACNA1C expression and diminished ICa-L current density. This mechanism contributes to shortened action potential duration, reduced calcium influx, and increased susceptibility to reentry and triggered activity, hallmarks of electrical remodeling in AF (11).

*In vivo*, IL-1β expression correlates with increased macrophage infiltration in the atria during both acute and chronic phases of AF (9). IL-1β not only downregulates QKI via NF-*κ*B-dependent transcriptional repression but also impairs QKI's ability to bind CACNA1C mRNA, thereby exacerbating the loss of channel expression. This axis is further potentiated by other stress-responsive signals including ROS and local hypoxia, which reinforce inflammatory signaling and contribute to the feed-forward cycle of electrical instability (33).

These findings indicate that macrophage-derived IL-1β suppresses QKI, resulting in downregulation of CACNA1C and impaired ICa-L function, thereby linking innate immune activation to cardiomyocyte electrophysiology. Targeting the IL-1β–QKI–CACNA1C axis may thus offer a promising immunomodulatory strategy to prevent electrical remodeling and AF perpetuation.

### 3.2 TREM-1–PI3 K/AKT–FoxO3a pathway

TREM-1 (Triggering Receptor Expressed on Myeloid Cells-1) is a member of the immunoglobulin superfamily expressed on the surface of monocytes and neutrophils (34). It activates downstream immune signaling pathways through interaction with DAP12, playing a central role as an amplifier of pro-inflammatory responses in various cardiovascular diseases (35, 36). In myocardial infarction and other inflammation-related cardiac pathologies, TREM-1 expression levels are significantly elevated and are associated with myocardial remodeling and poor outcomes (36).

TREM-1 is significantly upregulated in the atrial tissue of AF models and primarily localizes on infiltrated inflammatory macrophages. Upon activation, TREM-1 amplifies the inflammatory response by enhancing the release of proinflammatory cytokines and promoting the activation of the NLRP3 inflammasome. Mechanistically, TREM-1 engagement triggers the PI3 K/AKT signaling cascade. This activation leads to phosphorylation of AKT, which, in turn, phosphorylates the transcription factor FoxO3a, thereby altering its localization and transcriptional activity (35). The phosphorylated FoxO3a modulates the expression of a series of downstream inflammatory genes, thereby sustaining a proinflammatory state within the atrial myocardium (37). Notably, this cascade not only facilitates increased cytokine production but also favors further assembly of the NLRP3 inflammasome, which is a key mediator of sterile inflammation in AF (38). In models where atrial fibrillation is induced by Ang II, inhibition of TREM-1 markedly reduces macrophage infiltration, diminishes NLRP3 activation, and suppresses the overall inflammatory response, ultimately ameliorating electrical remodeling in the atria (35).

In summary, the TREM-1–PI3 K/AKT–FoxO3a pathway maintains a chronic inflammatory microenvironment in cardiac tissue by enhancing pro-inflammatory gene expression, downregulating anti-inflammatory signaling activity, and promoting immune cell recruitment in cardiac pathological states, which may provide a mechanistic basis for myocardial inflammation and fibrosis in diseases such as atrial fibrillation.

### 3.3 LIGHT–PI3K*γ*–SGK1 pathway

Tumor Necrosis Factor Superfamily Member 14 (LIGHT) is a member of the tumor necrosis factor (TNF) superfamily, primarily secreted by activated T cells and monocytes or macrophages (24, 39). It plays a crucial role in chronic inflammation and tissue remodeling. LIGHT activates multiple downstream signaling pathways through its receptor Herpesvirus Entry Mediator(HVEM) and has been found to significantly participate in myocardial remodeling and fibrosis in the cardiovascular system, particularly during the induction and maintenance stages of atrial fibrosis (24).

In the pathological process of atrial fibrillation, LIGHT activates SGK1 through a PI3K*γ*-dependent pathway (24). This pathway not only promotes the polarization of cardiac macrophages towards the M2 phenotype but also induces the activation and transdifferentiation of atrial fibroblasts, ultimately enhancing collagen synthesis and extracellular matrix deposition, thereby accelerating the formation of atrial fibrosis (24). The PI3K*γ*–SGK1 signaling axis is one of the core pathways in this process, and the activation of SGK1 is considered an important switch that converts inflammatory stimuli into a fibrotic phenotype (24). It promotes the metabolic activity and collagen transcription of fibroblasts by regulating targets such as FoxO and mTOR.

Beyond these upstream mechanisms, recent studies have provided direct experimental validation of the downstream actions of the LIGHT–PI3K*γ*–SGK1 axis. LIGHT stimulation increases PI3K*γ*-dependent phosphorylation of SGK1, which upregulates profibrotic genes including COL1A1, COL3A1, CTGF, and *α*-SMA, thereby enhancing fibroblast activation and extracellular matrix expansion (24). In primary cardiac fibroblasts, pharmacological inhibition or genetic knockdown of PI3K*γ* or SGK1 abolishes LIGHT-induced collagen production, confirming pathway specificity. Furthermore, *in vivo* studies have shown that elevated LIGHT expression correlates strongly with atrial fibrosis, and antibody-mediated LIGHT blockade reduces PI3K*γ*–SGK1 signaling and fibrosis burden in animal models (24).

Additionally, the LIGHT-mediated PI3K*γ*–SGK1 pathway may maintain the inflammatory microenvironment during the sustained phase of atrial fibrillation by inducing the expression of fibrotic factors such as TGF-*β*1, thereby further stabilizing the structural remodeling state of the atria (24, 40). In a chronic cardiac stress model, LIGHT levels were significantly elevated and positively correlated with the deposition levels of collagen I and III in the myocardium (41).

In conclusion, the TNFSF14/LIGHT–PI3K*γ*–SGK1 axis is not only an important mediator of the transition from chronic inflammation to fibrosis in the heart, but also provides a potential therapeutic target for atrial remodeling during the progression of atrial fibrillation.

### 3.4 NLRP3 inflammasome

The NLRP3 inflammasome, a key intracellular multiprotein complex, plays a pivotal role in the innate immune response and has been implicated in the pathogenesis of cardiac arrhythmias, including AF. Its activation requires two signals: a priming step, often mediated by NF-*κ*B, which increases the transcription of NLRP3 and pro-IL-1β, and a subsequent activation signal, such as ROS or mitochondrial damage, which induces inflammasome assembly and caspase-1 activation (42, 43).

In the context of AF, both cardiomyocytes and cardiac macrophages contribute to the pathological activation of the NLRP3 inflammasome, establishing a reciprocal inflammatory loop (36, 44). On one hand, damaged or stressed cardiomyocytes release damage-associated molecular patterns (DAMPs), including mitochondrial ROS, ATP, and oxidized DNA, which act as potent activators of NLRP3 in resident macrophages (45). Once activated, macrophages secrete IL-1β and IL-18, amplifying the inflammatory milieu and further sensitizing cardiomyocytes to electrical instability and structural remodeling. On the other hand, cardiomyocytes themselves also exhibit intrinsic inflammasome activation under stress conditions such as pressure overload or oxidative stress. For example, Al-Qazazi et al. demonstrated that in right ventricular failure associated with pulmonary arterial hypertension, NLRP3 activation in both cardiac macrophages and cardiomyocytes exacerbated mitochondrial dysfunction and contractile failure (43). Targeted inhibition of NLRP3 with MCC950 improved cardiac function and suppressed IL-1β release. These findings suggest a dual-cell contribution, where macrophage-derived inflammatory signaling triggers cardiomyocyte NLRP3 activation, which in turn releases more DAMPs, forming a vicious cycle (43).

Functionally, the downstream effects of inflammasome activation include pyroptosis, calcium-handling abnormalities, and gap junction disruption, all of which are closely linked to electrical remodeling in AF. IL-1β, in particular, has been shown to downregulate L-type calcium channel expression, promoting atrial conduction heterogeneity and reentry circuits (11). This aligns with the observation that chronic inflammasome activation correlates with atrial fibrosis and low-voltage zones in AF patients (46). Therefore, NLRP3 inflammasome activation within both macrophages and cardiomyocytes constitutes a critical mechanistic link between sterile inflammation and electrical remodeling in AF. Therapeutic blockade of this pathway, either via IL-1β neutralization or direct NLRP3 inhibition, holds translational promise in disrupting the pathogenic cross-talk and halting disease progression (42, 46). In addition to IL-1β, the inflammasome-related cytokine IL-18 also exerts significant electrophysiological effects on cardiomyocytes. IL-18 has been shown to promote Ca^2^^+^ handling abnormalities, enhance ROS generation, and increase vulnerability to triggered activity, thereby contributing to atrial arrhythmogenesis in inflammatory states (45, 47).

**Figure 2:**
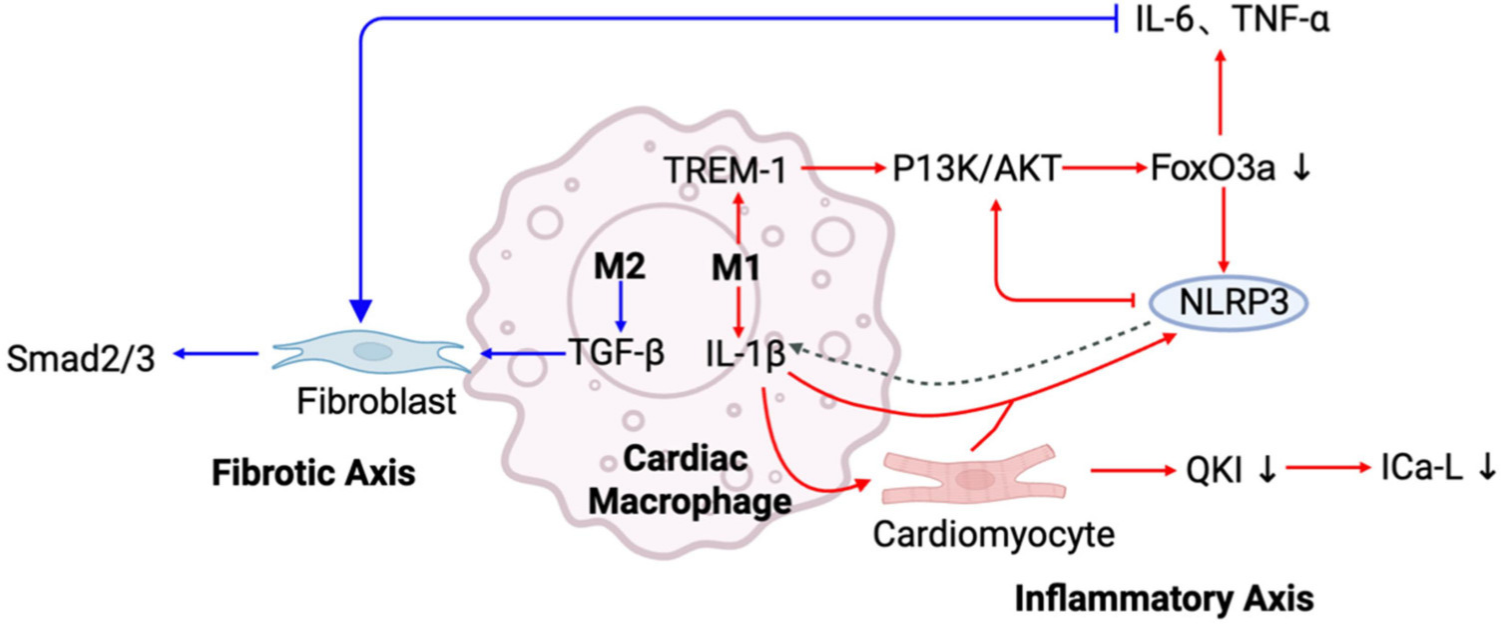
Inflammatory and fibrotic signaling axes mediated by cardiac macrophages in AF. This schematic summarizes two major signaling axes through which cardiac macrophages contribute to AF pathogenesis. In the inflammator*y* axis (red), M1 macrophages release IL-1β, activating TREM-1 and PI3 K/AKT signaling, which suppresses FoxO3a and enhances the expression of IL-6, TNF-α, and NLRP3 inflammasome activation. Activated NLRP3 further promotes IL-1β release in both macrophages and cardiomyocytes, forming a feed-forward inflammatory loop. IL-1β also impairs calcium handling in cardiomyocytes by downregulating QKI and ICa-L, contributing to electrical instability.

In summary, macrophages play key roles in both the inflammatory and fibrotic pathways through different subtypes and polarization (Table 1). The factors they secrete, such as IL-1β and TGF-*β*, drive the electrophysiological remodeling of cardiomyocytes and the activation of cardiac fibroblasts, leading to structural and functional abnormalities associated with atrial fibrillation (Figure 2). The aforementioned signaling axes also interact and regulate each other through positive feedback loops, forming a complex pathological network that supports the persistence of AF.

**Table 1:** Summary of Key macrophage-mediated signaling pathways in atrial fibrillation.

Pathway/Axis	Key Initiating Signal	Core Molecular Components	Primary Effectors & Downstream Targets	Main Pathological Outcome in AF
IL-1β–QKI–CACNA1C Axis	Macrophage-derived IL-1β	IL-1β → (NF-*κ*B) → QKI ↓ → CACNA1C mRNA destabilization	↓ L-type calcium current (I_Ca−L_) ↓ Action potential duration ↑ Triggered activity	Electrical Remodeling: APD shortening, conduction heterogeneity, increased reentry susceptibility
TREM-1–PI3 K/AKT–FoxO3a Pathway	TREM-1 activation on macrophages	TREM-1 → PI3 K/AKT → p-FoxO3a → NLRP3 inflammasome activation	↑ Pro-inflammatory gene expression ↑ NLRP3 inflammasome assembly ↑ IL-1β/IL-18 secretion	Inflammatory Remodeling: Sustained pro-inflammatory state, amplification of sterile inflammation, electrical instability
LIGHT–PI3K*γ*–SGK1 Pathway	LIGHT (from macrophages/T cells)	LIGHT → PI3Kγ → SGK1 → (FoxO/mTOR)	↑ M2 macrophage polarization ↑ Fibroblast activation (α-SMA) ↑ COL1A1, COL3A1, CTGF expression	Structural Remodeling: Fibroblast-to-myofibroblast transition, collagen deposition, atrial fibrosis
NLRP3 Inflammasome–ROS Signaling	DAMPs (ROS, ATP, mtDNA)	NLRP3 → ASC → Caspase-1 → pro-IL-1β/IL-18 → mature IL-1β/IL-18	Pyroptosis ↑ Active IL-1β & IL-18 Gap junction disruption Ca^2^^+^ handling abnormalities	Integrated Remodeling: Electrical instability (via cytokines) and structural changes (via inflammation-fibrosis crosstalk)

## 4 Functional impact of macrophage-driven remodeling on atrial substrate

Recent consensus definitions of atrial cardiomyopathy (ACM) underscore AF as a manifestation of progressive atrial structural, electrical, contractile, and molecular remodeling. ACM encompasses inflammatory, fibrotic, and metabolic alterations that precede AF onset (48). Integrating this concept strengthens the clinical relevance of macrophage-driven remodeling, as macrophage activation represents a central mechanism linking inflammation with fibrosis, conduction abnormalities, and atrial substrate degeneration (48). Beyond their immunological roles, cardiac macrophages exert profound structural and electrophysiological influences on the atrial substrate during AF development. Accumulating evidence has shifted the paradigm from viewing inflammation as a secondary bystander to recognizing immune cells, particularly macrophages, as active effectors that reshape the atrial microenvironment (49). Through the secretion of cytokines, matrix-modifying enzymes, and paracrine mediators, macrophages interact with resident atrial fibroblasts, cardiomyocytes, and endothelial cells, ultimately orchestrating both structural and electrical remodeling. These alterations not only sustain the arrhythmogenic substrate but also promote the transition from paroxysmal to persistent AF.

Macrophages in atrial tissue not only contribute to inflammation and fibrosis but also play critical roles in tissue repair and angiogenesis. Reparative macrophage subsets, induced by cytokines such as IL-4 and IL-10, secrete factors like VEGF and PDGF, promoting endothelial cell proliferation and tissue recovery (50–52). These functions help stabilize atrial tissue following injury, balancing the pro-inflammatory effects of macrophages. A dysregulation in this balance, with an overdominance of inflammatory phenotypes, may contribute to the pathological progression of AF.

### 4.1 Structural remodeling mediated by M2 macrophages

Atrial structural remodeling, a critical pathological substrate for AF, is characterized by fibroblast activation, excessive extracellular matrix (ECM) deposition, and progressive fibrosis (53). Among the multiple cellular contributors, M2-polarized macrophages play a pivotal role by facilitating the transformation of fibroblasts into myofibroblasts and promoting collagen deposition, which together drive fibrotic remodeling (54). M2 macrophages are typically induced under the influence of IL-4 and IL-13 and are marked by high expression of CD206, arginase-1, and TGF-*β*1. These cells secrete profibrotic cytokines, notably transforming growth factor-beta (TGF-*β*), which is central to activating fibroblasts and inducing their transdifferentiation into *α*-SMA + myofibroblasts (23, 25). This transition enhances the contractile and matrix-synthetic phenotype of fibroblasts, leading to accumulation of type I and III collagen and expansion of the interstitial space within the atrial wall.

Mechanistically, TGF-*β* released from M2 macrophages triggers canonical Smad2/3 signaling and promotes the upregulation of connective tissue growth factor (CTGF), further amplifying ECM synthesis and fibroblast proliferation (25, 55). Additionally, macrophage–fibroblast crosstalk may be reinforced by paracrine loops involving Galectin-3 and periostin, both of which are known to sustain fibrotic activity in cardiac tissue. In models of pressure overload and atrial stretch, the persistence of M2 macrophages correlates with increased atrial fibrosis and conduction heterogeneity (24). Notably, animal studies have demonstrated that depletion of M2 macrophages or inhibition of their TGF-*β* signaling axis attenuates atrial fibrosis and improves electrical stability.

Taken together, M2 macrophage-driven structural remodeling represents a key mechanistic axis by which inflammation is translated into sustained atrial substrate alteration. This fibrotic environment not only perpetuates AF but also impairs the response to rhythm control strategies, underscoring its clinical relevance.

### 4.2 Electrophysiological remodeling

Pro-inflammatory M1 macrophages play a pivotal role in promoting electrophysiological remodeling in AF, primarily through the secretion of cytokines such as IL-1β and TNF-α, which interfere with the expression and function of cardiac ion channels (17). These cytokines disrupt the homeostasis of calcium (Ca^2^^+^) and potassium (K^+^) currents, leading to a shortening of the atrial effective refractory period (AERP), thereby increasing the vulnerability of atrial cardiomyocytes to triggered activity and reentry mechanisms (11, 56). Specifically, IL-1β released by M1 macrophages has been shown to downregulate the L-type calcium channel subunit Cav1.2 via suppression of the RNA-binding protein QKI, leading to a reduction in Ica-L current density; this impairs action potential plateau formation and shortens AERP (6, 9, 11). Simultaneously, inflammatory conditions enhance the transient outward potassium current and inward rectifier potassium current, which further accelerate repolarization and facilitate reentry. This bidirectional crosstalk contributes to a pro-arrhythmic substrate by fostering action potential heterogeneity and promoting conduction discontinuities (57). In addition to ICa-L reduction, macrophage-driven inflammatory signaling can also influence other ion currents through ROS-mediated pathways. Cytokine-induced oxidative stress has been shown to enhance late Na^+^ current (INa,L) and modify repolarizing K^+^ currents, particularly IKr and IKs, thereby contributing to action potential heterogeneity and conduction slowing (58, 59).

Taken together, macrophage-driven inflammatory signaling orchestrates a cascade of ionic imbalances and electrical disturbances that are integral to the progression and maintenance of AF.

### 4.3 Interaction with fibroblasts

Macrophage-derived paracrine signaling plays a pivotal role in promoting atrial fibrosis by modulating fibroblast activation, proliferation, and ECM synthesis. Among these factors, TGF-β1 is a central profibrotic cytokine predominantly secreted by alternatively activated M2-like macrophages (18, 25, 60). TGF-β1 induces the phenotypic transition of quiescent cardiac fibroblasts into α-SMA^+^ myofibroblasts, thereby enhancing collagen I and III synthesis and contributing to excessive matrix deposition and atrial structural remodeling (41, 55). Additionally, IL-10, although primarily known for its anti-inflammatory properties, also participates in the fibrotic cascade. Evidence suggests that IL-10 supports fibroblast survival and can indirectly promote collagen accumulation through macrophage polarization into a TGF-β1-producing phenotype (13, 25). This suggests a context-dependent duality in IL-10's role, where prolonged IL-10–rich environments may foster fibrotic progression rather than resolution.

**Figure 3:**
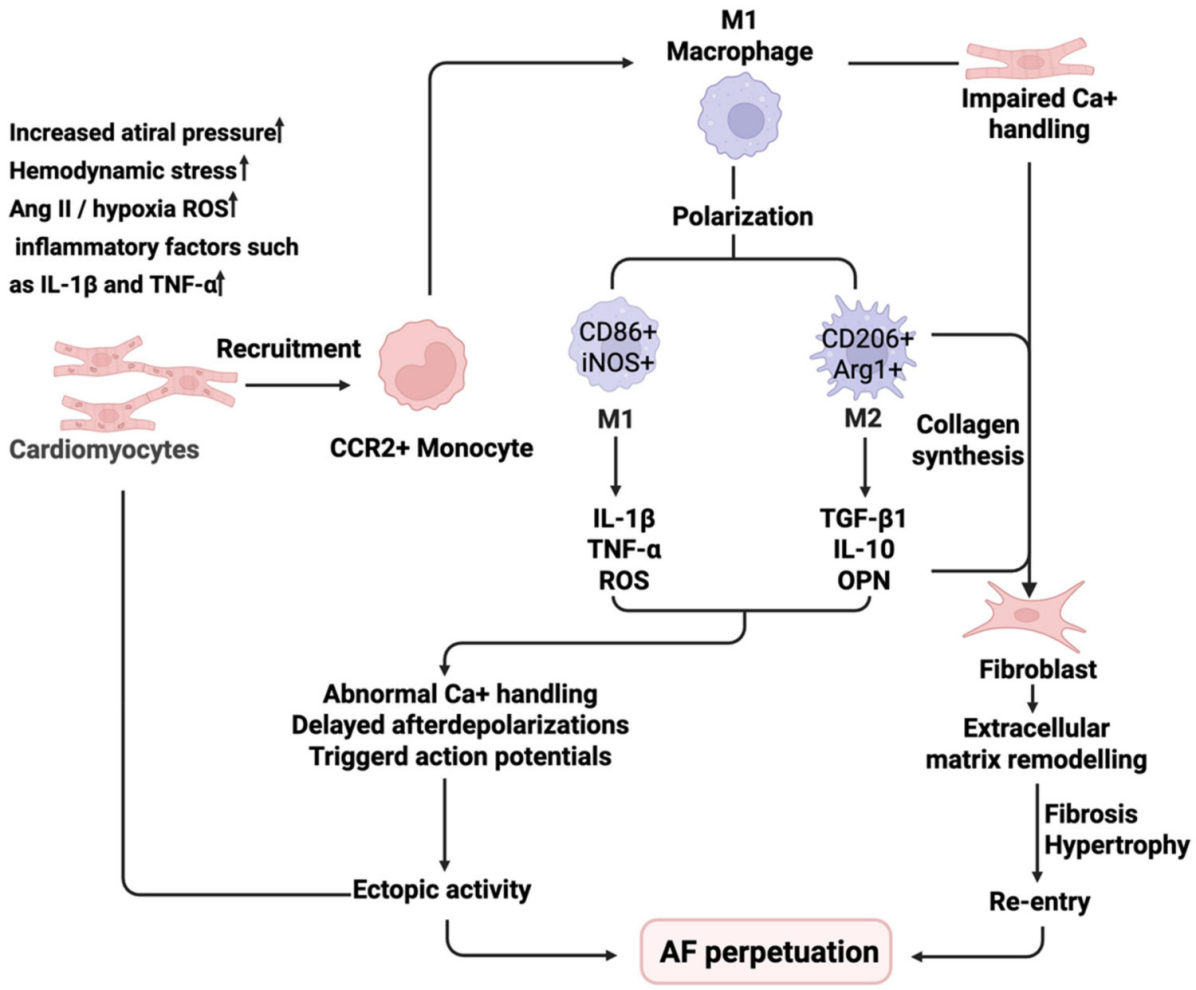
Cardiac macrophage recruitment, polarization and effector pathways in atrial fibrillation AF. Hemodynamic stressors (e.g., increased atrial pressure, Ang II, hypoxia, ROS, and local IL-1β/TNF-α) initiate the recruitment of CCR2^+^ monocytes from the circulation into atrial tissue. These monocytes differentiate into cardiac macrophages and undergo polarization into M1 (CD86^+^, iNOS^+^) or M2 (CD206^+^, Arg1^+^) subtypes. M1 macrophages secrete pro-inflammatory mediators (IL-1β, TNF-α, ROS), leading to impaired calcium handling, ectopic activity, and electrophysiological remodeling in cardiomyocytes. M2 macrophages promote collagen synthesis in fibroblasts through the release of TGF-*β*1, IL-10, and osteopontin (OPN), contributing to extracellular matrix remodeling, fibrosis, and structural remodeling. These processes form a feedforward loop that supports AF perpetuation.

The above content systematically elucidates the differential mechanisms underlying the activation and differentiation of macrophages, as well as the pro-inflammatory and pro-reparative pathways in atrial fibrillation, and their profound effects on the electrophysiological homeostasis of cardiomyocytes and the remodeling of the fibroblast-derived matrix.To clearly illustrate this multi-step, multi-pathway interactive process, a mechanism diagram (Figure 3) has been created to integrate cell recruitment, polarization, secretion of factors, and their role in the perpetuation of AF.

## 5 Evidence from animal models and clinical observations

### 5.1 Evidence from animal models

Animal model studies have provided important support for elucidating the mechanisms by which macrophages mediate inflammatory responses, structural, and electrophysiological remodeling in AF (61). In mice, dog, and rat models, a significant increase in the number of macrophages in atrial tissue is closely associated with AF susceptibility and persistence, often accompanied by fibrotic changes such as fibroblast activation and collagen deposition (14, 21, 22, 62).

In an angiotensin II-induced model, the study found significantly enhanced polarization of M2 macrophages in cardiac tissue, accompanied by upregulation of TGF-β signaling and increased activation of fibrosis-related markers such as fibroblasts and myofibroblasts, suggesting that macrophages may promote cardiac fibrosis through the TGF-β pathway (63, 64). Additionally, the LIGHT signaling pathway has been confirmed to regulate fibroblast activity and collagen synthesis in a mouse atrial fibrillation model, with LIGHT blockade significantly reducing fibrotic deposition and atrial fibrillation duration (24).

In C57BL/6 mice, IL-1β inhibits QKI expression in cardiomyocytes, reducing the mRNA stability of CACNA1C, leading to downregulation of ICa-L, and inducing electrical remodeling and increased susceptibility to atrial fibrillation (57). The use of IL-1 receptor antagonists or IL-1β gene knockout reversed these electrophysiological changes (65). Furthermore, activation of the NLRP3 inflammasome in an atrial fibrillation rat model has been shown to be closely associated with elevated IL-1β expression in myocardial tissue (46). In addition, in a rapid atrial pacing dog model, inflammatory cell infiltration, including increased macrophage presence, has been consistently observed, accompanied by the release of inflammatory factors such as IL-6 and TNF-α, as well as a shortened AERP, establishing the foundation for inflammatory–electrophysiological imbalance (21, 66). Further studies revealed that the PI3 K/AKT–FoxO3a pathway plays a central role in maintaining the pro-inflammatory phenotype of macrophages, and FoxO3a deficiency significantly reduces the expression of pro-inflammatory factors and improves electrophysiological stability (35, 40, 67).

Notably, multiple studies employing macrophage depletion strategies confirmed that removing macrophages in atrial fibrillation models significantly alleviates atrial structural remodeling and atrial fibrillation burden, further validating their decisive role in the pathological process (16, 22).

### 5.2 Clinical evidence

In clinical biopsy and pathological studies of atrial tissue in patients with atrial fibrillation, multiple pieces of evidence have been accumulated indicating that immune cell infiltration is closely associated with structural and electrophysiological remodeling (14, 68). In particular, the infiltration of M1-type macrophages has been shown to significantly increase in persistent AF, accompanied by elevated levels of IL-1β and NLRP3 expression, suggesting that the activation of inflammatory pathways plays a key role in atrial fibrillation (5).

In clinical data on fibrosis-related factors, TGF-*β*1 and LIGHT were significantly more highly expressed in atrial tissue from atrial fibrillation patients compared to sinus rhythm controls (24). Some studies confirmed that TGF-*β*1 is focally overexpressed in the left atrial interstitial region of atrial fibrillation patients, and its expression is closely correlated with the collagen I/III ratio, suggesting a direct driving role in atrial fibrosis progression (69, 70). Additionally, clinical sample analysis indicated that LIGHT is highly expressed in atrial fibrillation tissue and can activate the NF-*κ*B and Smad pathways, further enhancing fibroblast collagen secretion capacity (24). Furthermore, a study of serum and tissue samples from atrial fibrillation patients confirmed that elevated TREM-1 expression levels were positively correlated with inflammatory factor concentrations, and activation of its downstream PI3 K/AKT/FoxO3a pathway was also validated in clinical tissues, revealing that this pathway may participate in maintaining a pro-inflammatory microenvironment and promoting electrophysiological instability (35, 71).

Overall, these clinical observations validate the central role of macrophages and their mediated signaling pathways in atrial fibrillation from multiple dimensions, including histology, transcriptomics, and protein levels.

### 5.3 Omics evidence

With the rapid development of single-cell omics and spatial transcriptomics technologies, the heterogeneity, signal transduction activities, and intercellular interactions of macrophages in atrial fibrillation have been more systematically characterized (27, 72). In scRNA-seq analysis of human atrial tissue, researchers identified multiple functionally heterogeneous populations of cardiac immune cells, among which CD14^+^Ly6C^+^ mononuclear/macrophage cells were significantly enriched in atrial fibrillation patients and exhibited enhanced expression of inflammatory genes such as IL1B, NLRP3, and TNF, suggesting their central role in maintaining inflammatory environments and fibrotic remodeling (73).

In an integrated analysis of multimodal omics data, atrial tissue under inflammatory conditions exhibited highly reprogrammed transcriptional features, with downregulation of QKI, activation of NLRP3, and simultaneous activation of the FoxO3a pathway (74). Spatially, this manifested as the migration and accumulation of macrophages into the atrial endocardium and their aggregation in regions densely populated by fibroblasts. This local signal co-activation of the TGF-*β*/SMAD pathway further promoted the expansion of atrial fibrosis (28, 74, 75). Additionally, spatial transcriptomics revealed that the IL-1β signaling axis in atrial fibrillation tissue exhibits a band-like distribution along the atrial posterior wall and pulmonary vein entrance regions. Combined with immunofluorescence staining, this showed pronounced aggregation of CD68^+^IL-1β^+^ macrophages in these areas, accompanied by decreased CACNA1C expression and focal Connexin-43 disruption, suggesting its interference with local electrical conduction stability (76, 77).

Together, these findings from bulk and single-cell transcriptomics, proteomics, and spatial profiling studies converge on a core set of inflammatory and fibrotic mediators driven by distinct macrophage subpopulations in the atrial tissue. A summary of quantitative trends from representative animal, *in vitro*, and clinical studies is presented in Table 2, highlighting consistent increases in macrophage infiltration, pro-inflammatory signaling, fibrosis markers, and electrophysiological disturbances across models.

**Table 2:** Summary of quantitative findings from animal, clinical studies on macrophages in AF.

Study Type	Model/Species	Macrophage Marker	Macrophage Density	Markers	Electrophysiological Changes	Key Findings	References
Animal Model	Angiotensin II (Ang II)-induced model	M2 macrophages	Significant increase in M2 macrophages in atrial tissue	Increased TGF-β, fibroblast activation	Shortened action potential duration, increased susceptibility to AF	M2 macrophages promote fibrosis via TGF-β, enhancing fibroblast activation in AF	(63, 64)
Animal Model	IL-1β-induced model in C57BL/6 mice	IL-1β-expressing macrophages	Increased macrophage infiltration in atrial tissue	Decreased CACNA1C expression, reduced ICa-L current density	Shortened action potential, increased susceptibility to AF	IL-1β suppresses QKI, leading to decreased CACNA1C expression and electrophysiological remodeling	(57, 65)
Animal Model	NLRP3 inflammasome activation in AF rat model	NLRP3, IL-1β	Increased NLRP3 inflammasome activation	Increased IL-1β, IL-18	Calcium-handling abnormalities, reentry circuits	NLRP3 inflammasome activation in both macrophages and cardiomyocytes linked to atrial fibrosis and electrical instability	(46)
Animal Model	Rapid atrial pacing dog model	M1 macrophages	Increased macrophage infiltration in atrial tissue	Elevated IL-6, TNF-α	Shortened AERP	Inflammatory macrophage infiltration correlates with electrical remodeling and shortened AERP in AF	(21, 66)
Clinical Study	Atrial fibrillation patients (biopsy)	M1 macrophages	Increased infiltration in persistent AF	Elevated TGF-β1, LIGHT	Shortened AERP	M1 macrophage infiltration correlates with fibrosis and inflammatory response in AF	(5, 24, 68)
Clinical Study	Atrial tissue from AF patients	TGF-β1, LIGHT	High expression in atrial tissue	Collagen I/III ratio increased, fibroblast activation	Electrical remodeling, atrial fibrosis	TGF-β1 overexpression in left atrial interstitial region is associated with fibrosis progression	(69, 70)
Clinical Study	Atrial tissue from AF patients	TREM-1	Elevated TREM-1 expression	Increased NLRP3, TGF-β1	Increased susceptibility to electrical instability	TREM-1 correlates with inflammatory markers and the PI3 K/AKT/FoxO3a pathway in AF	(35, 71)

## 6 Therapeutic implications and future perspectives

### 6.1 Pharmacological interventions targeting macrophages and signaling pathways

Accumulating mechanistic evidence highlights the central role of macrophage polarization and secretory signaling in mediating atrial remodeling, providing a theoretical basis for therapeutic targeting(13). Inhibition of pro-inflammatory macrophage pathways, particularly those involving NLRP3 inflammasome activation, has emerged as a promising strategy. NLRP3 inhibitors such as MCC950 have shown efficacy in reducing atrial fibrosis and suppressing AF inducibility in preclinical rodent models by dampening IL-1β production and downstream fibrotic responses (5, 46). Besides MCC950, two other drug strategies targeting macrophages are also under investigation. One is CCR2/CCL2 axis inhibitors, which aim to block monocyte recruitment, thereby reducing the infiltration of pro-inflammatory macrophages in atrial tissue. Preclinical models show that CCR2 inhibition can alleviate myocardial fibrosis and adverse remodeling (19). The other is TGF-β signaling modulators, which target the interaction between macrophages and fibroblasts during fibrotic matrix formation. However, the clinical application of TGF-β blockers is hindered by their pleiotropic effects, although they can reduce fibrosis, systemic blockade may impair tissue repair or promote inflammation. Therefore, more refined targeting strategies are needed (49, 55).

On the other hand, targeting upstream regulators of macrophage-driven fibrosis, including the TGF-β1 and LIGHT pathways, also holds therapeutic potential. Experimental blockade of TGF-β signaling has been shown to attenuate fibroblast-to-myofibroblast transition and collagen deposition in atrial tissues, leading to reduced structural remodeling (55, 69). Meanwhile, LIGHT pathway modulation may interfere with lymphotoxin-like stimulation of collagen synthesis, although its translational relevance to AF remains underexplored. Additionally, anti-inflammatory interventions aiming to rebalance macrophage polarization, such as promoting the M2 phenotype or suppressing M1-type cytokine output, have yielded cardioprotective effects in other fibrotic cardiovascular conditions and may extend to atrial remodeling (3, 78).

Collectively, targeted modulation of macrophage activation states and their downstream effector pathways may offer novel and potentially synergistic avenues for the prevention or reversal of atrial fibrillation. However, further translational studies are required to evaluate the safety, timing, and specificity of such interventions in AF patients. Emerging biomaterial and nanocarrier platforms offer targeted modulation of macrophage activation in inflammatory cardiovascular disease. Some studies reported that nanoparticles preferentially internalised by macrophages enable precise delivery of inflammasome inhibitors or microRNA mimics (79). Although data in atrial fibrillation are limited, extension of these platforms to the atrial immune-substrate axis may provide second-generation immunotherapeutics.

### 6.2 Multi-dimensional combined intervention strategy

In atrial fibrillation, macrophage-mediated inflammation and fibrotic remodeling involve complex pathological mechanisms, including cell polarization, factor release, signaling pathway activation, and interactions between cardiomyocytes and fibroblasts. Single-target interventions often fail to achieve sustained therapeutic effects. Therefore, developing a multi-dimensional combined intervention strategy has become an important direction for future precision treatment of atrial fibrillation (53, 80).

On one hand, existing studies have demonstrated that targeting different pathways simultaneously can produce synergistic effects (57, 81). On the other hand, clinical sample omics analyses have revealed heterogeneity in patients' immune inflammation, metabolic status, and pathway activation levels, suggesting that intervention strategies should be tailored to individual patients and involve multi-pathway combination (27, 74, 82).

Currently, the combined approaches of interest include inhibition of inflammatory factors combined with regulation of polarization states, pathway inhibition combined with RNA regulation, and inhibition of fibroblast activation combined with electrophysiological co-regulation (3, 78). However, several clinical trials involving TGF-β have failed, primarily because TGF-β plays a dual role in both fibrosis and repair (55). Currently, more precise interventions, such as blocking potential TGF-β activation, targeting downstream signaling pathways, or time-limited dosing are under investigation.

In summary, multidimensional combined interventions should not only focus on synergistic inhibition of signaling pathways but also integrate factors such as intervention timing, tissue targeting, and pharmacokinetics in their design.

### 6.3 Individualized immune reprogramming paradigms

Emerging evidence from multi-omics studies and spatial transcriptomics reveals significant heterogeneity in macrophage activation states, immune signaling profiles, and structural remodeling phenotypes among AF patients (27, 28, 74, 82). This heterogeneity underpins the limited efficacy of uniform therapeutic regimens and highlights the need for individualized immune reprogramming paradigms tailored to patient-specific immunopathological features.

At the core of this strategy lies precise immune phenotyping, which integrates data from bulk RNA-seq, scRNA-seq, and multiplex immunohistochemistry to classify AF patients into distinct immuno-structural endotypes. For example, a fibro-inflammatory subtype is characterized by abundant M2 macrophage infiltration, TGF-β–SMAD axis activation, and diffuse atrial fibrosis, whereas a pro-arrhythmic inflammatory subtype features dominant M1 polarization, elevated IL-1β and NLRP3 expression, and prominent electrophysiological instability (82). Building on this framework, immune reprogramming can be designed to selectively modulate key cellular and molecular targets. In the fibro-inflammatory subtype, therapeutics such as IL-10 antagonists, anti-TGF-β antibodies, or LIGHT signaling inhibitors may be prioritized to suppress fibrosis and restore homeostasis. In contrast, for the pro-arrhythmic inflammatory subtype, strategies focusing on IL-1β blockade, NLRP3 inflammasome inhibition, and QKI pathway restoration may be more effective in mitigating conduction abnormalities (80). Recent evidence emphasises that biological sex influences macrophage behaviour and atrial remodeling in AF. For example, the sex-specific differences in atrial electrophysiology were highlighted by a review, hormonal regulation and immune responses in AF, suggesting that gender-dependent macrophage phenotypes may modify atrial substrate vulnerability (83). Incorporating sex-stratified macrophage profiles may thus enhance personalised immunomodulatory strategies in AF. However, suppressing fibrosis completely is not advisable as reparative fibrosis is essential for tissue stability and healing (84). In early atrial remodeling, fibrosis plays a reparative role, helping stabilize tissue and prevent rupture. In later stages, however, pathological fibrosis can exacerbate the condition (85). Therefore, timing of intervention is critical. Early-stage therapies may promote beneficial fibrosis, while later-stage treatments should focus on limiting excessive fibrosis and restoring electrical stability.

### 6.4 Future perspectives

Despite accumulating insights into the role of macrophages in AF, substantial gaps remain between mechanistic discoveries and effective clinical translation. Moving forward, several key research directions are critical for advancing macrophage-centered therapeutic strategies and integrating them into the broader landscape of AF management.

First, spatiotemporal profiling of macrophage subsets during different AF stages remains underexplored. While cross-sectional studies have identified M1/M2 imbalances in atrial tissues, longitudinal tracking using fate-mapping or intravital imaging is essential to delineate the dynamic transitions between macrophage phenotypes and their temporal relationship with structural and electrical remodeling.

Second, multi-cellular crosstalk mechanisms, particularly between macrophages, fibroblasts, endothelial cells, and cardiomyocytes, should be dissected using integrative platforms such as spatial transcriptomics, single-cell multi-omics, and organoid co-culture systems. These approaches can reveal context-specific ligand-receptor interactions, paracrine loops, and transcriptional networks that drive atrial remodeling.

Third, the development of macrophage-targeted delivery systems remains in its infancy. Nanoparticle-mediated siRNA delivery, antibody-conjugated carriers, and macrophage-tropic viral vectors offer promising avenues for selectively modulating inflammatory pathways or reprogramming polarization in atrial tissue. However, challenges such as bioavailability, off-target effects, and long-term immunogenicity require careful investigation.

Fourth, future clinical studies should move toward precision immunomodulation trials, incorporating patient stratification based on immune phenotypes and remodeling burden. Biomarker panels including circulating monocyte and macrophage markers, serum cytokines, and imaging-based fibrosis metrics may help identify responsive subgroups and monitor therapeutic response longitudinally.

Lastly, safety and ethical considerations must accompany innovation. As immune interventions carry the risk of systemic immunosuppression or exacerbation of autoimmunity, robust preclinical safety data, careful patient selection, and long-term outcome monitoring are essential to ensure clinical applicability.

## 7 Conclusion

AF is increasingly recognized not solely as an electrophysiological disorder, but as a complex disease driven by immune-mediated structural and electrical remodeling. Among immune cell populations, macrophages play a pivotal role in orchestrating the inflammatory milieu, mediating fibroblast activation, modulating cardiomyocyte electrophysiology, and bridging the gap between immune signaling and atrial substrate remodeling.

This review highlights those macrophages, through phenotypic polarization, paracrine factor secretion, and cellular interactions, contribute to key pathophysiological processes including atrial fibrosis, conduction heterogeneity, and reentrant circuit formation. Interventions targeting macrophage activation, polarization, and associated signaling pathways hold promising therapeutic value. Advances in delivery systems, immunophenotyping, and omics-based profiling open new avenues for precision-guided, mechanism-oriented AF treatments. By systematically integrating current knowledge from *in vitro* experiments, animal models, clinical observations, and multi-omics analyses, this review provides a comprehensive framework for understanding macrophage-mediated mechanisms in AF. These findings lay the groundwork for future translational research and innovative therapies aimed at immune modulation and atrial remodeling prevention in AF.
